# DNA Tweezers with Replaceable Clamps for the Targeted Degradation of Cell Membrane Proteins

**DOI:** 10.3390/pharmaceutics17060785

**Published:** 2025-06-17

**Authors:** Yang Sun, Yichen Huang, Daiquan Chen, Shangjiu Hu, Tao Pan, Yuanding Liu, Ruowen Wang, Weihong Tan

**Affiliations:** 1 Shanghai Key Laboratory for Nucleic Acid Chemistry and Nanomedicine, Institute of Molecular Medicine (IMM), Renji Hospital, School of Medicine, Shanghai Jiao Tong University, Shanghai 200127, China; suny1989@sjtu.edu.cn (Y.S.); hyichen19@sjtu.edu.cn (Y.H.);; 2 Zhejiang Cancer Hospital, Hangzhou Institute of Medicine (HIM), The Chinese Academy of Sciences, Hangzhou 310022, China

**Keywords:** nucleic acid delivery, aptamer, LYTACs, cancer treatment, membrane protein, targeted degradation

## Abstract

**Background**: Cell membrane proteins play crucial roles in signal transduction and nutrient transport. Many membrane proteins are reportedly overexpressed in cancer cells, which is closely related to cancer progression. The targeted degradation of these membrane proteins has been demonstrated to be a promising strategy for tumor treatment. Several strategies using aptamers to mediate membrane protein lysis, such as lysosomal-mediated lysis and proteasome-mediated lysis, have been reported, but their efficiency is limited by the binding affinity of the aptamer to a single target. **Methods**: We constructed DNA tweezers with replaceable clamps, which can lyse different proteins upon clamp replacement. Moreover, the clamp improved the degradation efficiency of the target proteins by enhancing the specificity and improving the binding affinity. **Results**: Lysis was verified in different tumor cell lines and the antitumor activity was confirmed in zebrafish. **Conclusions**: Overall, these DNA tweezers improve the efficiency of the targeted delivery of functional nucleic acids, provide an efficient and versatile strategy for the degradation of disease-causing proteins, and expand the approach to antitumor therapy.

## 1. Introduction

Cell membrane proteins play crucial roles in biological functions such as cell-to-cell interactions, signal transduction, and the membrane transport of ions or small molecules [1]. Given their cell surface presence, they are usually the first to be affected by external signals, especially in a tumor microenvironment, and their various expression levels lead to irreversible cellular stress responses and malignant transformation, resulting in cancer emergence and progression [2]. In addition, cancer and noncancer cell lines exhibit different mutation and post-translational modification patterns [3]. These factors make cell membrane proteins major cancer biomarkers and drug targets; although they represent only 30% of the human genome, they account for 60–70% of drug targets. Drugs, including small-molecule inhibitors and monoclonal antibodies, can inhibit tumor cells by targeting the active sites of certain tumor-associated membrane proteins and blocking their ligand–receptor interactions [4]. However, protein mutations can lead to tyrosine kinase inhibitor (TKI) resistance, and inactive targets that lack clear ligand binding pockets are considered undruggable [5]. In addition, high concentrations of small-molecule inhibitors must persist to achieve therapeutic effects (occupancy-driven pharmacology), which leads to the compensatory upregulation of disease proteins and increased toxicity [6].

Targeted protein degradation is a promising therapeutic strategy that utilizes the proteasome or lysosomal system to selectively degrade proteins that are closely associated with a disease, offering considerable advantages over inhibitors in cancer therapy. Protein hydrolysis-targeting chimeras (PROTACs) and molecular glues that rely on the ubiquitin proteasome system are already present in clinical trials [7]. However, PROTACs mostly degrade cytoplasmic proteins, and their ability to degrade cancer-associated cell membrane proteins is limited [8]. The lysosome-targeting chimera (LYTAC) strategy is an emerging technology in which an antibody targets a protein and a ligand fragment of a cell surface lysosomal transport receptor (LTR), which transports the target protein to the lysosome for degradation [9,10,11]. Aptamer-based LYTAC strategies have recently been reported. Aptamers, identified by the systematic evolution of ligands via exponential enrichment (SELEX), are a class of short RNA or DNA oligonucleotides that bind specifically to their target molecules (e.g., proteins, peptides, or even living cells) [12,13]. In 2021, Han et al. [14] synthesized the first bispecific DNA aptamer-based LYTAC, which consists of a DNA aptamer that binds tightly to IGFIIR (Insulin-like growth factor-2 receptor) and a DNA aptamer that binds to a cell membrane receptor through a junction sequence, which effectively targets and induces the degradation of cell membrane proteins. Since then, several studies have improved and refined the aptamer-based LYTAC strategy [15,16,17].

The binding affinity of LYTAC systems for their target proteins and the ability of the complexes to be internalized influence protein degradation [18]. In addition to their excellent binding affinity and internalization ability, aptamers, such as nucleic acids, can also be used as building blocks for structurally tunable nanomaterials, such as DNA tweezers, which have two clamps for capture [19]. Bivalent and multivalent aptamers exhibit increased binding affinity to their target [20,21]. as well as a better stability [22,23]. At present, DNA tweezers have been utilized in biosensing, drug delivery, and molecular manipulation applications. Nevertheless, their application in protein degradation has not been reported [24,25,26].

In this study, we report on the use of DNA tweezers for the targeted degradation of cell membrane proteins. The arm of the DNA tweezers is an IGFIIR-targeted aptamer, while the two clamps are aptamers that target the membrane proteins of interest; additionally, different membrane proteins can be degraded by replacing the clamps. NCL (Nucleolin), a protein that is abnormally highly expressed on the surface of many kinds of tumor cells, was chosen for study, along with the AS1411 aptamer that specifically binds to NCL [27]. Compared with the aptamer alone, the DNA tweezers incorporating AS1411 were associated with a greater stability, increased binding affinity, and improved internalization of the aptamer chimeras, showing excellent degradation of NCL. The antitumor efficacy of the DNA tweezers was also evaluated in vivo. Degradation was also observed for EGFR- and PDL1-targeting systems when the clamps were replaced. Finally, protein degradation by the DNA tweezers was studied with respect to time and concentration.

## 2. Materials and Methods

### 2.1. Chemicals, Cell Lines, and Reagents

DNA sequences (Table S1), DNA staining dye Gel-red, Mg (Ac)_2_, Trisborate EDTA buffer, 30% polyacrylamide, TEMED, and APS (Ammonium persulphate) were from Sangon Biotech (Shanghai, China). DEPC (Diethyl Pyrocarbonate) water, Dulbecco’s PBS, yeast tRNA, BSA, glucose, and agarose were from Hongsheng Biotech (Shanghai, China). Anti-GAPDH antibody (sc-137179) was purchased from Santa Cruze (Starr County, TX, USA). Nucleolin antibody (14574T) was from Cell Signaling Technology (Danvers, MA, USA). EGFR antibody (4267T) was from Cell Signaling Technology. Na^+^-K^+^-ATPase antibody (sc-21713) was from Santa Cruze. PDL1 antibody (14-5983-82) was from Thermo Fisher (Waltham, MA, USA). Ki67 antibody (#9129) was from Cell Signaling Technology. Caspase-3 antibody (#9664) was from Cell Signaling Technology. Alexa Fluor 488-Nucleolin antibody(ab202708) was from Abcam (Cambridge, UK). Goat Anti-Rabbit IgG (Alexa Fluor 488) (ab150077) was from Abcam. Goat Anti-Mouse IgG (Alexa Fluor 488) (ab150113) was from Abcam. LysoTracker Green was from Shanghai Maokang Biotechnology (Shanghai, China). MCF-7 cells were cultured in DMEM with 10% fetal bovine serum at 37 °C under a 5% CO_2_ atmosphere. Hela cells were cultured in DMEM with 10% fetal bovine serum at 37 °C under a 5% CO_2_ atmosphere. The 4T1 cells were cultured in RPMI 1640 medium with 10% fetal bovine serum at 37 °C under a 5% CO_2_ atmosphere. Washing buffer was used to remove excess aptamers post incubation with cells and was made by mixing 4.5 g/L glucose and 5 mM MgCl_2_ in Dulbecco’s PBS with calcium chloride and magnesium chloride. Binding buffer was used for the binding of aptamers with cells and was prepared by adding yeast tRNA (0.1 mg/mL; Sigma-Aldrich, St. Louis, MO, USA) and BSA (1 mg/mL; Fisher Scientific, Hampton, NH, USA) in washing buffer to reduce non-specific binding.

### 2.2. Construction of DNA Tweezers and Mo-AS in Buffer

For each pair of DNA tweezers, nucleic acid module 1 and nucleic acid module 2 were mixed and dissolved in ultrapure water at a concentration of 1:2. The DNA mixture solution was annealed as follows: 95 °C for 10 min, 65 °C for 30 min, 50 °C for 30 min, and 37 °C for 30 min. A 12% native polyacrylamide gel in 1 × TBE buffer was run at 120 V for 90 min and was then stained with Gel Red staining solution.

### 2.3. Serum Stability Test

A total of 1 μm of DNA tweezers and Mo-AS was incubated with 10% fetal bovine serum (FBS) for different lengths of time (1–24 h). A 12% native polyacrylamide gel in 1 × TBE buffer was run at 120 V for 90 min at 4 °C, before being stained with Gel Red staining solution.

### 2.4. Flow Cytometry Analysis of Aptamer Binding to MCF-7 Cells

To determine the binding affinities of aptamer chimeras to cells, MCF-7 cells were incubated separately with a series of different concentrations of AS1411, DNA tweezers, and Mo-AS in 100 μL of binding buffer on ice for 30 min. The cells were washed twice with washing buffer, centrifuged at 1000 rpm for 3 min, and then resuspended in 200 μL of binding buffer. The labeled cells were analyzed on CytoFLEX (Beckman Coulter Life Sciences, Brea, CA, USA) by counting 10,000 events. FlowJo software (V 10.0.8r1) was used for data analysis. The mean fluorescence intensity of the cell–aptamer complexes was used to evaluate the binding affinity. The dissociation constants (Kds) of aptamer chimeras were obtained by fitting the dependence of fluorescence intensity (Y) and the concentrations of aptamer (X) into the one-site saturation equation Y = Bmax X/(Kd + X), using GraphPad Prism 7.0; Bmax represents the maximum binding capacity.

### 2.5. Lysosomal Colocalization Experiment Using Confocal Microscope

MCF-7 cells cultured in confocal Petri dishes were co-incubated with LysoTracker Green (Maokang Biotechnology, Shanghai, China) at 37 °C for 1 h. Cells were then washed twice with wash buffer, followed by the addition of 250 nM Cy5-labeled AS1411, DNA tweezers, and Mo-AS for varying incubation times at 37 °C. The cells were then washed twice with washing buffer. Cells were imaged on a Leica confocal fluorescence microscope (Leica Biosystems, Hessen, Germany). Throughout the experiment, a 638 nm He-Ne laser was the excitation source for Cy5, while a 488 nm UV laser was the excitation source for LysoTracker.

### 2.6. Detection of Cell-Surface Nucleolin by Flow Cytometry

MCF-7 cells were plated in 12-well plates (2 × 10^5^ cells per well) before the experiment. The cells were exposed to various DNA tweezers at different concentrations (0, 50, 150, 250, 500, and 750 nM) for either 24 h or specific time intervals, as specified. Subsequently, the cells were enzymatically dissociated into a single-cell suspension. Cells were then resuspended in 100 μL of DPBS solution containing 5% BSA and incubated with Alexa Fluor^®^ 488-labeled nucleolin monoclonal antibody (Cell Signaling Technology, Danvers, MA, USA) for 30 min (in an ice bath). After incubation, the cells were washed, resuspended in cold PBS, and analyzed using flow cytometry to detect labeled cells, wherein 10,000 events were counted.

### 2.7. Detection of Cell-Surface Nucleolin by Western Blot Analysis

Cellular membrane proteins were isolated through a subcellular fractionation approach utilizing the Membrane Protein Extraction Kit (Beyotime Biotechnology (Shanghai, China), Cat. No. P0033). The cell lysate was centrifuged and the supernatant was collected. The protein concentration was determined using the BCA (Bicinchoninic Acid Assay) protein assay kit (Thermo Fisher Scientific, Waltham, MA, USA). Proteins were separated via 10% SDS-PAGE and transferred onto a polyvinylidene fluoride (PVDF) membrane (Millipore, Burlington, MA, USA). Membranes were blocked with 5% skimmed milk for 1 h at room temperature and were then incubated with primary antibodies (1:1000) overnight at 4 °C. After being washed three times with TBST (Tris-Borate-Sodium Tween-20, Beyotime Biotechnology, Shanghai, China), membranes were incubated with secondary antibodies (1:5000) for 1 h at room temperature. Membranes were washed three times with TBST and were incubated with an ECL (electrochemiluminescence) chemiluminescent substrate (Bio-Rad, Hercules, CA, USA). A quantitation of band intensities was performed using Image-Lab 6.1 Software (Bio-Rad Laboratories, CA, USA).

### 2.8. Detection of Cell-Surface EGFR by Flow Cytometry

MCF-7 cells were plated in 12-well plates (2 × 10^5^ cells per well) before the experiment. The cells were incubated with 250 nM of EGFR-DNA tweezers for 24 h (optimized conditions). Subsequently, the cells were enzymatically dissociated into a single-cell suspension. Cells were then resuspended in 100 μL of DPBS solution containing 5% BSA and were incubated with EGFR monoclonal antibody for 30 min (in an ice bath), and then with Alexa Flour 488 goat Anti-Rabbit IgG for 30 min. After incubation, cells were washed, resuspended in cold PBS, and labeled cells were detected by flow cytometry, wherein 10,000 events were counted.

### 2.9. Western Blot Analysis

Cells were lysed with RIPA (Radio Immunoprecipitation Assay) buffer containing phenylmethanesulfonyl fluoride (PMSF) and phosphatase inhibitor cocktail on ice for 30 min. The cell lysate was centrifuged and the supernatant was collected. The protein concentration was determined using the BCA (Bicinchoninic Acid Assay) protein assay kit (Thermo Fisher Scientific). Proteins were separated via 10% SDS-PAGE and were transferred onto a polyvinylidene fluoride (PVDF) membrane (Millipore). Membranes were blocked with 5% skimmed milk for 1 h at room temperature and were then incubated with primary antibodies (1:1000) overnight at 4 °C. After being washed three times with TBST (Tris-Borate-Sodium Tween-20), membranes were incubated with secondary antibodies (1:5000) for 1 h at room temperature. Membranes were washed three times with TBST and were incubated with an ECL (electrochemiluminescence) chemiluminescent substrate (Bio-Rad). A quantitation of band intensities was performed using Image Lab 6.1 Software (Bio-Rad Laboratories, CA, USA).

### 2.10. In Vivo Imaging

All animal operations were in accord with institutional animal use and care regulations, approved by the Renji Hospital Laboratory Animal Center. Four-week-old female CB17 nude mice (Shanghai Laboratory Animal Co., Ltd., Shanghai, China) were given a subcutaneous injection of 1 × 10^7^ MCF-7 cells into the backside of the right hind. Cy5-labeled AS1411, Mo-AS, or DNA tweezers were intravenously injected (15 μM, 100 μL) when the tumor size reached 150 mm^3^, after mice were anesthetized with a tranquilizer and the anesthetic isopentane. Images were obtained at certain timepoints. The tumors and major organs (heart, kidney, liver, spleen, and lung) were collected for ex vivo imaging with an IVIS Lumina XP (PerkinElmer, Waltham, MA, USA) imaging system.

### 2.11. Immunohistochemistry

Tumor tissues were fixed in 10% neutral-buffered formalin, embedded in paraffin, and sectioned at 4–5 μm thickness. Sections were deparaffinized, rehydrated, and subjected to antigen retrieval. Endogenous peroxidase activity was blocked, followed by incubation with primary antibodies against NCL, Ki67, and Caspase-3. After washing, sections were incubated with secondary antibodies, followed by streptavidin–HRP (Horseradish Peroxidase) and DAB (Diaminobenzidine) substrate for color development. Then, sections were scanned with the Digital Pathology Slide Scanner (Leica biosystems, Hessen, Germany)

### 2.12. Animal Care and Husbandry

Wild-type AB-strain zebrafish and the transgenic green fluorescent Flk1 strain zebrafish were provided by Hunter Biotechnology Co. Ltd. (Hangzhou, China), and the feeding management met the requirements of AAALAC international certification (001458). All protocols were approved by the Animal Ethics Committee of the Institute (no. SYXK (Zhe) 2022-0004) [28]. Embryos were incubated with fish water at 28 °C and collected after natural mating.

### 2.13. Xenograft Zebrafish Model

MCF-7 cells, labeled with a green fluorescent Dio (3,3′-dioctadecyloxacarbocyanine perchlorate) cell-labeling solution (Thermo Fisher Scientific, Waltham, MA, USA), were microinjected into the yolk sac of wild-type AB-strain zebrafish embryos at 2 days postfertilization (dpf). Approximately 200 cells were transplanted into each zebrafish tail, creating a zebrafish breast cancer xenograft model. Following the injection, these xenograft-bearing larvae were carefully maintained at a constant temperature of 25 °C throughout the duration of the experiment.

## 3. Results and Discussion

### 3.1. Synthesis and Characterization of the DNA Tweezers

**Scheme 1:**
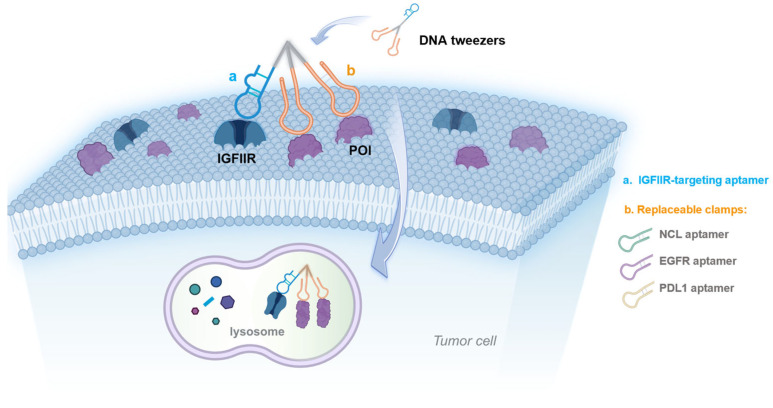
Schematic of DNA tweezers with replaceable clamps for the lysosomal degradation of target proteins.

**Figure 1:**
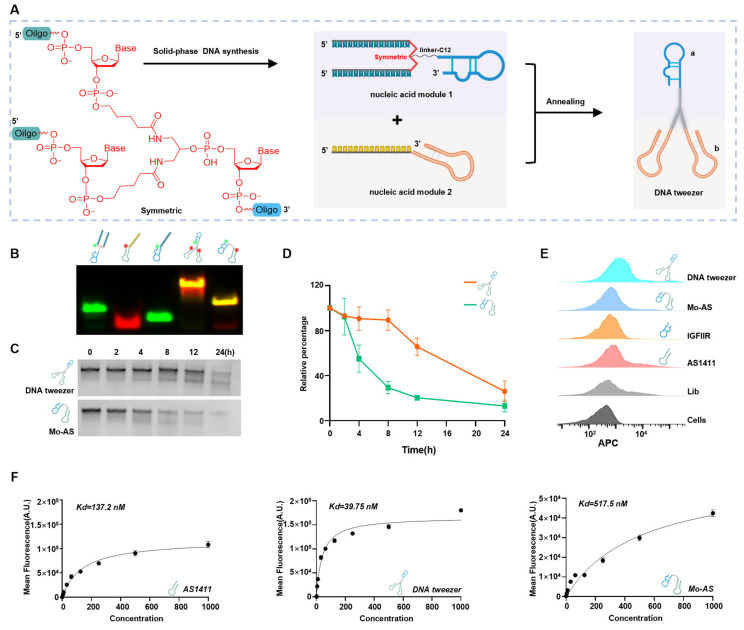
Characterization of the DNA tweezers. (**A**) Strategy through which the DNA tweezers were synthesized. The black wavy line represents the linker-C12. ‘a’ represents IGFIIR-targeting aptamer, ‘b’ represents replaceable clamps. (**B**) Native polyacrylamide gel electrophoresis of the aptamer chimeras. From left to right, Lane 1: FAM-labeled module 1; Lane 2: Cy5-labeled module 2 (target NCL); Lane 3: FAM-labeled polyT-IGFIIR; Lane 4: Cy5-, FAM-labeled DNA tweezers (target NCL); Lane 5: Cy5-, FAM-labeled Mo-AS. (**C**) DNA tweezers and Mo-AS were incubated with culture medium supplemented with 10 % FBS for certain durations, and the stability of each was studied via native polyacrylamide gel electrophoresis. (**D**) The gray values were quantified, and the degradation curves of the DNA tweezers and Mo-AS are shown. The data are shown as the means ± SDs (*n* = 3). (**E**) The binding ability of DNA tweezers to MCF-7 cells was examined via flow cytometry. Library (a control sequence composed of randomly synthesized N nucleotides (A, T, C, G) that serves as a negative control in aptamer-based experiments), AS1411, IGFIIR aptamer, and Mo-AS were used as controls. (**F**) Analysis of the binding affinity of AS1411, DNA tweezers, and Mo-AS for MCF-7 cells.

Symmetric, which is a branching treble phosphoramidite base containing three protecting groups onto which three nucleic acid chains can be synthesized, was used to construct the DNA tweezers (Scheme 1 and Figure 1A). The IGFIIR-targeted aptamer and a C12 linker (a linear alkyl chain configuration comprising twelve carbon atoms) were introduced at the 3′ end of Symmetric through DNA solid-phase synthesis. An oligonucleotide sequence consisting of 15 thymine residues (T15) was introduced at both 5′ ends for clamp assembly, yielding nucleic acid module 1. Module 2 (the clamps) was composed of an aptamer for the proteins of interest and a chain composed of 15 adenine residues (A15). Nucleic acid modules 1 and 2 were assembled onto the DNA tweezers at a 1:2 ratio through complementary base pairing. Additionally, replacing the clamps allows for the lysosomal degradation of different proteins. All nucleic acid sequences were synthesized by Shanghai Sangon biotech (Shanghai, China). The DNA sequences used in this study are shown in Table S1.

The inclusion of two clamps should provide a bivalent effect; to confirm this, a monovalent aptamer chimera Mo-AS (Figure S1) consisting of a single heterodimeric aptamer was used as a control. Native polyacrylamide gel electrophoresis (native-PAGE) was first performed to verify the formation of the DNA tweezers, and the slower migration of higher-molecular-weight DNA tweezers was observed (Figure 1B), demonstrating the successful assembly of the clamps and arms. The distinct electrophoretic bands were spectroscopically assigned as follows: green fluorescence corresponds to 5-carboxyfluorescein (FAM)-conjugated oligonucleotides, red emission designates cyanine 5 (Cy5)-labeled strands, and the observed orange-yellow chromatic signatures correspond to co-localized fluorophores on individual oligonucleotide strands. Multichannel fluorescence imaging was achieved through wavelength-specific excitation (λ_ex = 488 nm and 635 nm for FAM and Cy5, respectively) coupled with spectral unmixing algorithms. The Cy5 or FAM fluorophore was covalently conjugated to the 5′-terminus of the aptamer (FAM labeled-module1; Cy5 labeled-module2; FAM-polyT-IGFIIR; Nucleic acid module 2 (target NCL); Cy5-Nucleic acid module 1; Cy5-Nucleic acid module 2 (target NCL); Cy5-Nucleic acid module 2 (target EGFR); Cy5-Nucleic acid module 2 (target PDL1); Cy5-Library) using a commercially available labeling service. Specifically, by using a DNA solid-phase synthesis machine, the aptamer was synthesized with a 5′-terminal amine modification during solid-phase synthesis. The Cy5-NHS or FAM-NHS (N-hydroxysuccinimide) ester was then reacted with the primary amine group under mild alkaline conditions, forming a stable amide bond. Then, the stability of the DNA tweezers was studied by incubation in Dulbecco’s modified eagle medium (DMEM) containing 10% fetal bovine serum (FBS) at 37 °C for different durations. The DNA tweezers were detectable until 12 h, while Mo-AS clearly degraded by the 4 h timepoint (Figure 1C,D). Next, the binding ability and affinity of the DNA tweezers with AS1411 clamps were studied via flow cytometry with breast cancer cells—MCF-7—which have been reported to highly express NCL and have moderate IGFIIR protein levels [29]. The results revealed that both AS1411 and IGFIIR bound to the cells and that the DNA tweezers displayed a stronger binding affinity (Figure 1E). The binding affinities of the DNA tweezers applied at different concentrations were subsequently determined (Figure S2A–C). We found that the cellular equilibrium dissociation constants of AS1411, the DNA tweezers, and Mo-AS were 137.2 nM, 39.75 nM, and 517.5 nM, respectively, (Figure 1F). As for Mo-As and AS1411, we speculate that AS1411 inherently exhibits a strong binding affinity to cells, while the IGFIIR nucleic acid aptamer has a weaker binding affinity to its target; the Mo-AS complex may exist by dynamically binding to IGFIIR or/and NCL, which shows an improved Kd value. Compared with a single aptamer, the monovalent aptamer chimeras showed an even weaker binding affinity to the cells, whereas the bivalent aptamer chimeras showed the highest binding affinity, which may be attributable to the greater structural stability and bivalent effect of the DNA tweezers.

### 3.2. Internalization and Lysosomal Colocalization of the DNA Tweezers

**Figure 2:**
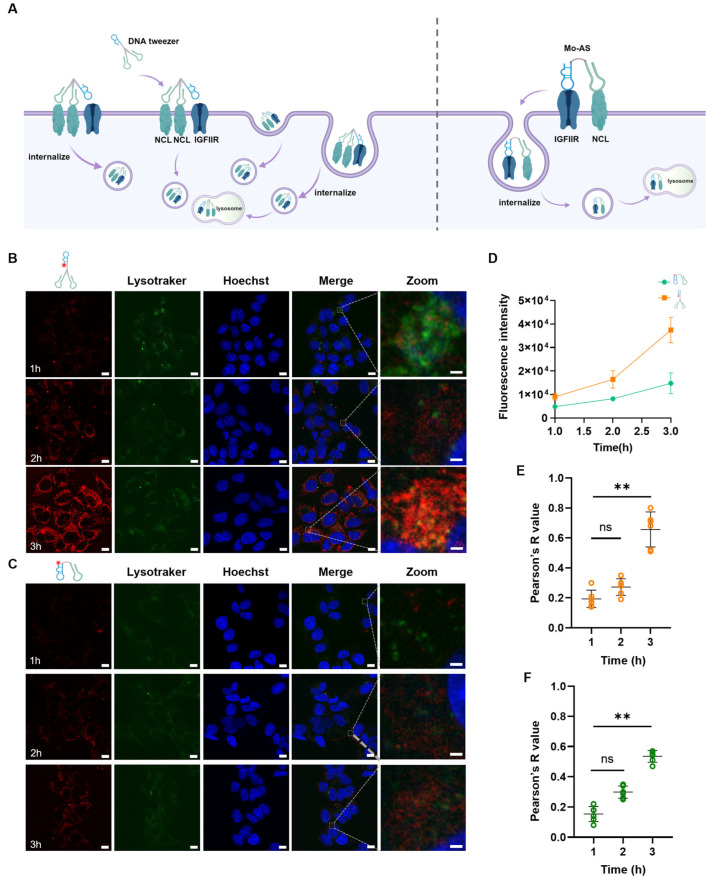
Internalization of the DNA tweezers. (**A**) Schematic of the cellular internalization and lysosomal colocalization of DNA tweezers and Mo-AS. (**B**) Confocal images showing the cellular uptake and lysosomal localization of DNA tweezers in MCF-7 cells. Scale bar: 10 μm and 1 μm. (**C**) Confocal images showing the cellular uptake and lysosomal localization of Mo-AS in MCF-7 cells. Scale bar: 10 μm and 1 μm. (**D**) The fluorescence intensity of intracellular DNA tweezers and Mo-AS at different times. The data are shown as the means ± SDs (*n* = 3). (**E**) Colocalization of DNA tweezers and lysosomes determined via Pearson’s correlation from three independent experiments. The data are shown as the means ± SDs (*n* = 6). ** *p* < 0.01; ns, not significant. (**F**) Colocalization of Mo-AS and lysosomes determined via Pearson’s correlation from three independent experiments. The data are shown as the means ± SDs (*n* = 6). ** *p* < 0.01; ns, not significant.

Internalization is a prerequisite for lysosome degradation; considering the enhanced binding affinity of the DNA tweezers to cells, we next examined their internalization efficiency and lysosome colocalization (Figure 2A). Cy5-labeled DNA tweezers were incubated with MCF-7 cells at 37 °C for different durations (1 h, 2 h, and 3 h). Additionally, the lysosomes were labeled with LysoTracker Green and the nuclei were labeled with Hoechst 33342 (blue); the cells were subsequently visualized via confocal microscopy. As shown in Figure 2B, the DNA tweezers were internalized by MCF-7 cells within 1 h, and the internalization efficiency increased with increasing incubation duration, peaking at 3 h. The clear colocalization of the DNA tweezers with lysosomes was also observed at 3 h. The internalization efficiency (Figure 2C,D) and lysosome colocalization in the Mo-AS group were significantly lower than those in the DNA tweezer group (Figure 2E,F). Taken together, these data suggest that the bivalent effect of the DNA tweezers promoted aptamer chimera uptake and accelerated their translocation to lysosomes.

### 3.3. DNA Tweezer-Mediated Degradation of Membrane NCL

**Figure 3:**
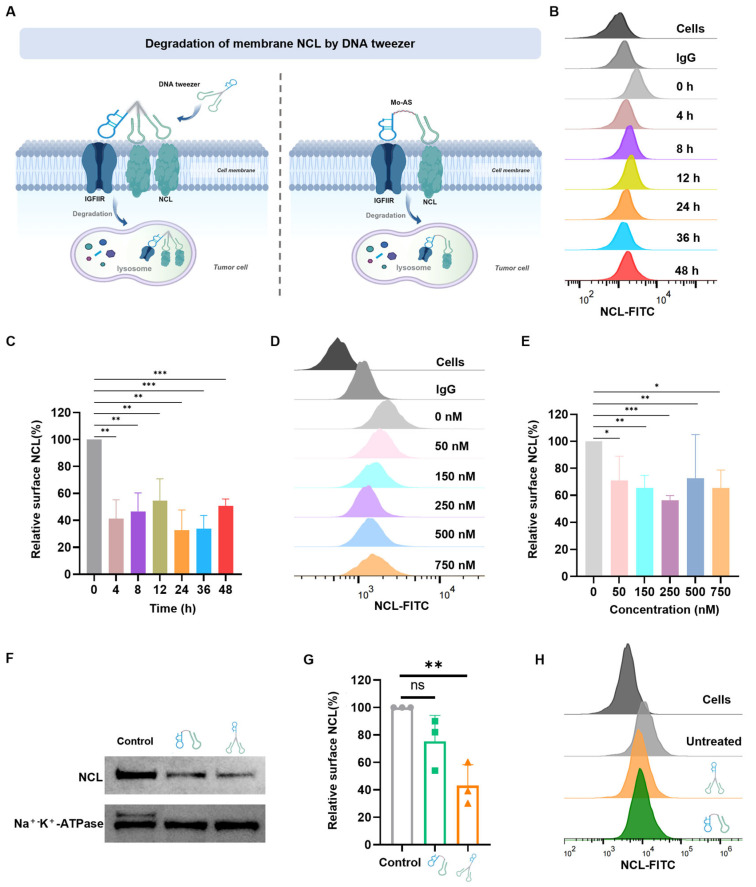
Degradation of membrane NCL by DNA tweezers. (**A**) Schematic of NCL degradation by DNA tweezers and Mo-AS. (**B**) DNA tweezers were incubated with MCF-7 cells for different durations, and the amount of NCL on the cell surface was determined via flow cytometry with an Alexa Fluor 488-anti-NCL antibody. (**C**) Quantification of the fluorescence intensity in (**B**). The data are shown as the means ± SDs (*n* = 3). ** *p* < 0.01, *** *p* < 0.001. (**D**) MCF-7 cells were incubated with different concentrations of DNA tweezers for 24 h, and the concentration of NCL on the cell surface was determined via flow cytometry. (**E**) Quantification of the fluorescence intensity in (**D**). The data are shown as the means ± SDs (*n* = 3). * *p* < 0.05, ** *p* < 0.01, *** *p* < 0.001. (**F**) NCL protein levels on the cell surface after treatment with DNA tweezers or Mo-AS at a concentration of 250 nM for 24 h were studied by Western blotting. (**G**) The gray value of each band was quantified in (**G**). The data are shown as the means ± SDs (*n* = 3). ** *p* < 0.01; ns, not significant. (**H**) NCL protein level on the cell surface was examined via flow cytometry with an anti-NCL antibody after treatment with DNA tweezers or Mo-AS.

Through the internalization and lysosome transport of the DNA tweezers, protein degradation can be achieved. Thus, the DNA tweezers (250 nM) were incubated with MCF-7 cells for 0–48 h, and the NCL protein expression on the cell surface was analyzed by flow cytometry with an anti-NCL antibody. The results revealed that the protein level of NCL fluctuated with time, with the first degradation peak appearing at 4 h, and approximately 70% of the NCL protein was degraded by 24 h (Figure 3B,C); nonetheless, an inverse relationship was observed between the incubation duration and the resulting degradation efficiency, which might be due to the renewal mechanism of membrane proteins. Furthermore, NCL degradation increased with increasing DNA tweezer concentration within a certain range over 24 h of incubation (Figure 3D,E). The peak degradation effect was observed with 250 nM DNA tweezers, and degradation efficiency decreased at higher DNA tweezer concentrations. This inverse concentration–response relationship may be attributed to the hook effect [30], where supraphysiological tweezer concentrations promote the formation of non-productive binary complexes (e.g., NCL-DNA tweezers or IGFIIR-DNA tweezers) rather than functional ternary complexes (NCL-DNA tweezers-IGFIIR). Furthermore, favorable or repulsive interactions between the protein of interest (POI) and IGFIIR may affect the formation of the NCL protein-DNA tweezers-IGFIIR protein ternary complex [31].

With the optimized incubation duration and DNA tweezer concentration determined, the degradation efficiency of DNA tweezers was further studied, with Mo-AS as a control. Cell membrane lysates were prepared after the cells were incubated with DNA tweezers or Mo-AS, and Western blot (WB) experiments were performed. The results (Figure 3F,G) revealed that both aptamer chimeras effectively induced protein degradation, but the degradation efficiency of DNA tweezers was approximately 30% greater than that of the monovalent aptamer chimeras. Then, flow cytometry was performed to verify the status of membrane NCL degradation (Figure 3H and Figure S3), and we observed more effective protein degradation in the DNA tweezer group. “Cells” represents the weak autofluorescence of the blank cell set, and “untreated” represents the NCL protein level (detected with anti-NCL antibody) on the surface of MCF-7 cells without drug treatment. The results above suggest that DNA tweezers have the advantage of the so-called handle effect, which enhances the binding and internalization of chimeras to cells and allows for the binding of multiple target proteins at the same time for transport to lysosomes [32], ultimately improving the efficiency of protein degradation. Notably, the degradation efficiency fluctuated with time and DNA tweezer concentration.

### 3.4. In Vivo Targeting and Biodistribution of the DNA Tweezers

**Figure 4:**
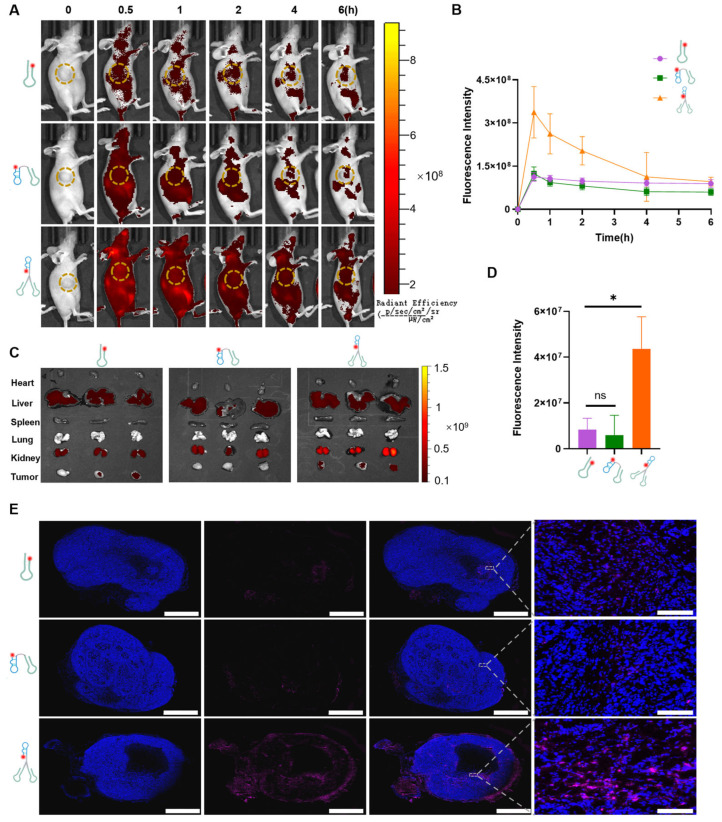
In vivo and ex vivo biodistribution of the DNA tweezers. (**A**) MCF-7 tumor-bearing mice were intravenously injected with Cy5-labeled AS1411, Mo-AS, or DNA tweezers, and images were captured at certain timepoints (*n* = 3 mice per group). The tumor was labeled with yellow circles. (**B**) Fluorescence intensities of DNA tweezers and Mo-AS in the circled region of the tumor (yellow) at 0, 0.5, 1, 2, 4, and 6 h. The data are shown as the means ± SDs (*n* = 3). (**C**) The biodistribution of AS1411, Mo-AS, and DNA tweezers in different organs. (**D**) Fluorescence intensities in isolated tumors 6 h after treatment with AS1411, Mo-AS, and DNA tweezers. The data are shown as the means ± SDs (*n* = 3). * *p* < 0.05; ns, not significant. (**E**) Tumor tissues were collected and tissue sections were prepared for confocal imaging. Nuclei stained with DAPI are shown in blue, and the Cy5-labeled aptamer appears in violet. Scale bar: 2000 μm and 100 μm.

To promote the application of the DNA tweezers, the in vivo targeting effect and biodistribution of the DNA tweezers were studied. Therefore, Cy5-labeled AS1411, Mo-AS, or DNA tweezers (15 µM, 100 µL) was injected into model mice bearing MCF-7 xenograft tumors via the tail vein, and fluorescence images were captured at different timepoints (Figure 4A). The AS1411 and Mo-AS groups presented similar fluorescence intensities at 0.5 h, which were weaker than that of the DNA tweezer group. The fast clearance of Mo-AS was also observed, whereas the DNA tweezers presented a better stability (Figure 4B). The major organs and tumors of the mice were collected 6 h after drug injection and were visualized (Figure 4C), and the fluorescence intensity was quantified (Figure 4D). Compared with the other two groups, the DNA tweezer group presented significantly stronger fluorescence signals. All three groups presented fluorescence enrichment in the liver and kidney, which may be related to the metabolism of the nucleic acids. However, differences in fluorescence intensity were observed at the tumor sites in the different groups. Additionally, tumor tissue sections were prepared to study penetration (Figure 4E and Figure S4). Purple fluorescence signals were clearly observed in the DNA tweezer group, which was consistent with the above results. Thus, these data demonstrate that the DNA tweezers have significantly enhanced in vivo stability and tumor-targeting effects, which are essential for rapid protein degradation.

### 3.5. In Vivo Interference Effect of DNA Tweezers on NCL and Its Antitumor Efficacy

**Figure 5:**
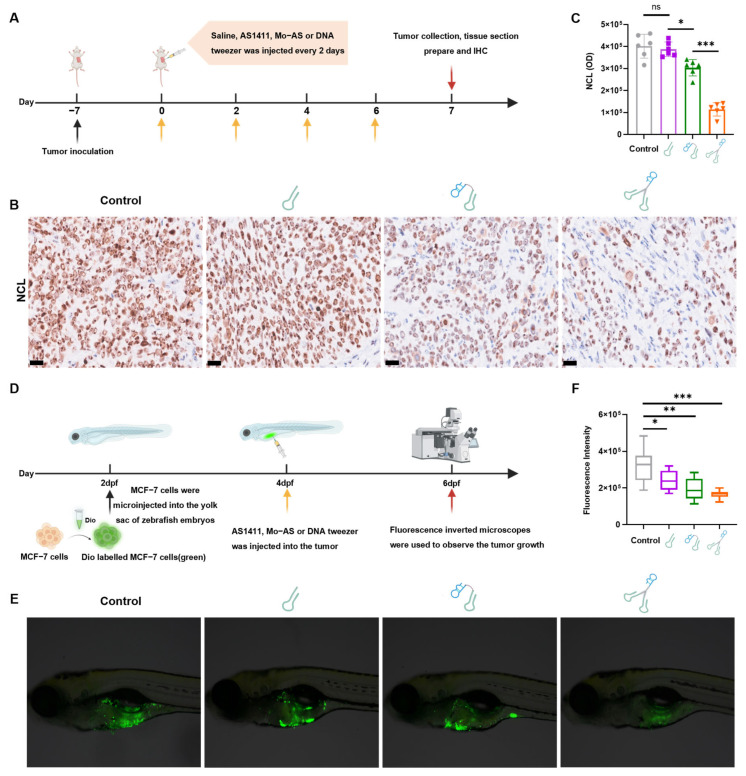
In vivo degradation effect of DNA tweezers on NCL. (**A**) Schematic illustration for the degradation procedure. MCF-7 cells were injected subcutaneously into mice. Saline was administered as a negative control. Image created with Biorender.com, with permission. (**B**) IHC was performed with anti-NCL antibody in tumor tissues. Scale bar: 25 μm. (**C**) Quantification of the optical density values of NCL in tumor cells (*n* = 3 tumors per group). For quantification, an analysis of a total of two fields per tumor was performed. * *p* < 0.05, *** *p* < 0.001; ns, not significant. (**D**) Schematic illustration for the treatment procedure in the zebrafish. (**E**) Green fluorescence represents tumor cells in zebrafish embryos under fluorescence microscopy at 48 h postinjection. (**F**) Quantification of total densities from tumor cells in zebrafish embryos using ImageJ software. The data are shown as mean ± SD (*n* = 10). * *p* < 0.05, ** *p* < 0.01, *** *p* < 0.001.

To evaluate the in vivo degradation potential of DNA tweezers, we established MCF-7 xenograft models in mice (Figure 5A). When the tumor size reached approximately 200 mm^3^, DNA tweezers were intratumorally injected every two days, three times (at a dosage of 2 mg/kg body weight, with individual dosing volumes adjusted to 40 μg per 20 g of mice). The control group injected with saline. Seven days later, tumor tissues were collected for analysis. The protein level was studied with immunohistochemical (IHC) staining. A significant weaker stain of anti-NCL was observed in the tumor tissues of the DNA tweezer group (Figure 5B,C), with a protein degradation efficiency exceeding twice that of the Mo-AS group, suggesting that DNA tweezers maintain sustained degradation activity in vivo, which results from their enhanced metabolic stability, improved internalization, and better tumor-targeting properties.

NCL on the cell surface was report to interact with FAS (Factor-related Apoptosis) and other membrane proteins, thereby promoting tumor cell proliferation and suppressing apoptosis [33]. We next tested the protein level of the proliferation marker Ki67 and the apoptosis marker caspase-3 in tumor tissues using IHC. The results showed that (Figure S5) DNA tweezers significantly increased cell death (Figure S5B) and robustly inhibited tumor cell proliferation (Figure S5C) compared with the AS1411 and Mo-AS groups. Given the superior degradation efficacy and anti-proliferative effects generated by DNA tweezers in vivo, we further explored their potential in tumor therapeutics.

Considering the significant molecular and pathological similarities between zebrafish and human cancer models, as well as the optical transparency of zebrafish that allows for the direct observation of tumor cell behavior in vivo [34], we established a zebrafish breast cancer model to investigate the antitumor therapeutic effects of DNA tweezers. MCF-7 cells were transplanted into wild-type AB-strain zebrafish (2 dpf); then, Cy5-labeled AS1411, Mo-AS, or DNA tweezers, which emit red fluorescence, were intravenously injected and the fish were maintained at 25 °C. After 0, 6, and 48 h, ten zebrafish were randomly selected from each group, and the distribution of the samples within the zebrafish was assessed using fluorescence microscopy. As depicted in Figure S6, the DNA tweezer group presented an increased Cy5 fluorescence intensity and tumor colocalization. Subsequently, we explored the antitumor efficacy of DNA tweezers; zebrafish were randomly assigned to four groups, (control, AS1411, Mo-AS, and DNA tweezers), with 30 tails in each group, and the appropriate sample (100 µM, 10 nL) was injected into the tumor site. After 48 h, ten zebrafish were randomly selected from each group for fluorescence microscopy evaluation (Figure 5D). As shown in Figure 5E, the green fluorescence intensity (indicating cancer cells) was significantly lower in the DNA tweezer group than in the other groups, indicating the therapeutic superiority of DNA tweezers. Additionally, the cancer cell travel distance toward the tails of the zebrafish was shortest in the DNA tweezer group than the other groups (Figure S7B). The quantitative analysis using ImageJ 2.0 software revealed a significant decrease in both the total density of cancer cells and the tail transfer distance following treatment with DNA tweezers (Figure 5F and Figure S7C). More importantly, both DNA tweezers and Mo-AS were more effective than AS1411 for tumor treatment, demonstrating that protein degradation exerts a more direct and potent effect in promoting tumor cell apoptosis and diminishing metastasis.

### 3.6. Generality of Using DNA Tweezers for Protein Degradation

**Figure 6:**
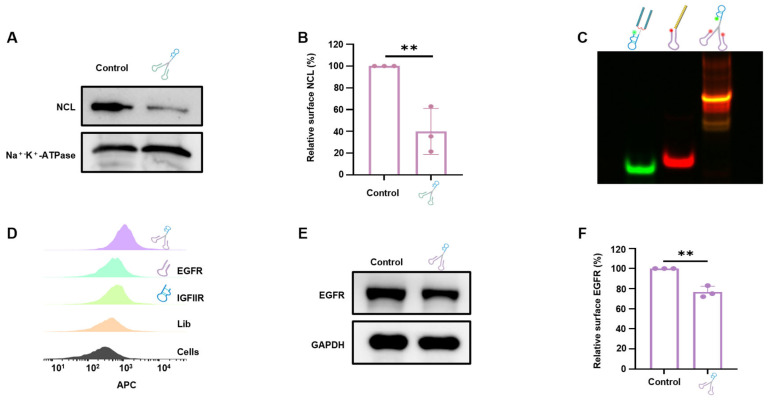
The universality of DNA tweezers in the targeted degradation of different membrane proteins with the corresponding clamps. (**A**) Degradation of NCL in 4T1 cells with NCL-targeted clamps was analyzed via Western blotting. (**B**) The gray value of each band was quantified. The data are shown as the means ± SDs (*n* = 3). ** *p* < 0.01. (**C**) The assembly of the EGFR-targeted DNA tweezers was verified via native polyacrylamide gel electrophoresis. From left to right, Lane 1: FAM-labeled module 1; Lane 2: Cy5-labeled module 2 (target EGFR); Lane 3: Cy5-, FAM-labeled DNA tweezers. (**D**) The binding ability of the EGFR-targeted DNA tweezers to Hela cells was analyzed via flow cytometry. Library, IGFIIR aptamer, and EGFR aptamer were used as controls. (**E**) EGFR protein levels were quantified by Western blotting following treatment with EGFR-targeting DNA tweezers. (**F**) The gray value of each band was quantified in (**E**). The data are shown as the means ± SDs (*n* = 3). ** *p* < 0.01.

The NCL in mice and humans shares 80% homology and similar structures [35,36], and the IGFIIR gene exhibits over 85% homology between mice and humans [37,38]. The functional domains of the encoded proteins are also remarkably similar; therefore, aptamers that bind targets on the basis of the 3D structure can bind NCL and IGFIIR from both species. Thus, 4T1 cells that highly express both NCL and IGFIIR were selected for verification [15,39]. DNA tweezers (250 nM) were incubated with the cells for 24 h, and then the cell membrane proteins were extracted for WB analysis. As illustrated in Figure 6A,B, DNA tweezers degraded approximately 50~80% of the NCL on the cell, which was similar to the percent degraded in MCF-7 cells, proving the advantage of using aptamers for target binding.

Next, clamps targeting different proteins were synthesized to examine the potential of DNA tweezers to degrade other membrane proteins. Epidermal growth factor receptor (EGFR), a known transmembrane glycoprotein that is overexpressed in various cancer cells and is essential for cancer cell proliferation, was chosen as a target [40]. The assembly of the EGFR-targeted DNA tweezers was first confirmed through native-PAGE (Figure 6C). Then, the binding affinity of DNA tweezers to EGFR-positive Hela cells was studied (Figure 6D), and protein degradation was subsequently analyzed by Western blotting (Figure 6E,F). The internalization effect was further studied with confocal microscopy; the results (Figure S8) show efficient cellular internalization (red) and lysosome colocalization (green), indicating the transport to the lysosome, which leads to EGFR degradation. We also examined the behavior of EGFR-DNA tweezers in MCF-7 cells, which have lower levels of IGFIIR and EGFR on the cell membrane. Both the flow cytometry (Figure S9B,C) and WB results (Figure S9D) revealed that the EGFR-DNA tweezers could effectively degrade EGFR on the MCF-7 cell surface.

The generality of these DNA tweezers was further demonstrated by the construction of PDL1-targeting DNA tweezers to regulate programmed death ligand 1 (PD-L1) (Figure S10A); specific binding, internalization, and notable degradation were observed (Figure S10B–D). “Control” represents the group without drug treatment. Taken together, these data suggest the universality of using these DNA tweezers to degrade different proteins in different species.

## 4. Conclusions

In this study, we developed DNA tweezers with replaceable clamps as a general protein degradation platform. This strategy has several advantages. First, DNA tweezers with two clamps provide bivalent effects, which improves the binding affinity of the aptamer to its target, thus improving internalization and resulting in better protein degradation. Notable protein degradation was detected in the experiments targeting NCL, EGFR, and PDL1. Second, considering the homology of proteins among different species, aptamers are less sensitive to species differences than antibodies are, as aptamers bind to their targets on the basis of the target structure. Obvious protein degradation effects were observed in human and mouse cancer cells. Third, the DNA tweezers with a lysosomal targeting aptamer as the arm and different clamps targeting different proteins can easily degrade proteins via the lysosome, as lysosome colocalization was visualized and analyzed, which may be extendable to PROTACs and autophagy-targeted chimeras (AUTACs) if aptamers are available. Moreover, we found that protein degradation was not linearly correlated with the incubation time of the DNA tweezers, as regular fluctuations were observed, which may be related to the renewal of the cytomembrane protein. The degradation effect was also not related to the concentration of the DNA tweezers, which should be explored in future research. Finally, and most importantly, the in vivo degradation ability of DNA tweezers was verified in mice, and antitumor activity was proven in zebrafish, indicating the potential of this degradation system for cancer treatment.

Leveraging its modular design, structure-specific targeting capability, and multi-pathway compatibility, DNA tweezer technology holds promise as a broad-spectrum antiviral platform in the future. By targeting essential viral replication proteins and integrating aptamers directed at the lysosomal or ubiquitin–proteasome pathways, this approach enables the specific degradation of viral proteins. In the biomanufacturing sector, coupling DNA tweezer systems with biosensors could establish a “sense-degrade” feedback loop, thereby optimizing bio-synthetic efficiency.

## Data Availability

The original contributions presented in this study are included in the article/Supplementary Material. Further inquiries can be directed to the corresponding authors.

## Supplementary Materials

The following supporting information can be downloaded at https://www.mdpi.com/article/10.3390/pharmaceutics17060785/s1. Figure S1: Synthetic strategy of Mo-AS. Figure S2: Binding affinity of AS1411 (**A**), DNA tweezers (**B**), and Mo-AS (**C**) to MCF-7 cells was studied with flow cytometry. Figure S3: MCF-7 cells were treated with DNA tweezers or Mo-AS for 3 h. The protein level of NCL was studied with flow cytometry, and the fluorescence intensity change was concert into protein concentration change. Figure S4: MCF-7 tumor-bearing mice were intravenously injected with Cy5-labeled AS1411, Mo-AS, or DNA tweezers. Tumor tissues were collected and tissue sections were prepared for confocal imaging. The intensity of residual Cy5 fluorescence on tumor tissue is shown for each group. Figure S5: (**A**) IHC stain of Ki67 and Caspase-3 in dissected tumor tissues. (**B**) Quantification of Ki67-positive cells in tumors. (**C**) Quantification of the optical density values of Caspase-3 in tumors. Figure S6: (**A**) Schematic illustration for exploring the distribution of AS1411, DNA tweezers, and Mo-AS in zebrafish over time. (**B**) MCF-7 tumor xenograft zebrafish model was constructed and Cy5-labeled AS1411, Mo-AS, and DNA tweezers (red) were injected intravenously. Then, imaging was performed with fluorescence microscopy. Tumor cells were labeled with green fluorescence. (**C**) Fluorescence values for each group on tumor sections. Figure S7: (**A**) Schematic illustration for exploring the inhibition of tumor (green) metastasis in zebrafish by AS1411, Mo-AS, or DNA tweezers. (**B**) Representative image of the metastatic distance of tumor cells to the tail 48 h post-injection. (**C**) Transfer distance of MCF-7 cells in the tail of zebrafish embryos 48 h after injection. Figure S8: Confocal images showing cellular uptake and lysosomal localization of EGFR-DNA tweezers in Hela cells. Figure S9: (**A**) Binding ability of EGFR-DNA tweezers to MCF-7 cells was tested with flow cytometry. (**B**) Cellular surface EGFR was studied with anti-EGFR antibody by flow cytometry after treatment with EGFR-DNA tweezers. (**C**) The fluorescence intensity of EFGR antibody was quantified. (**D**) EGFR protein level in MCF-7 cells after treatment with EGFR-DNA tweezers was verified by Western blotting. Figure S10: (**A**) The assembly of PDL1 targeted DNA tweezers was verified with native polyacrylamide gel. (**B**) Binding ability of PDL1-DNA tweezers to 4T1 cells was tested with flow cytometry. (**C**) Confocal images showing cellular uptake and lysosomal localization of PDL1-DNA tweezers in 4T1 cells. (**D**) PDL1 protein level of 4T1 cells after treatment with PDL1-DNA tweezers was verified by Western blotting. Figure S11: The structure of C12. Table S1. DNA sequences used in this work.

## Abbreviations

The following abbreviations are used in this manuscript:

LYTACs	Lysosome-targeting chimeras
IGFIIR	Insulin-like growth factor-2 receptor
NCL	Nucleolin
dpf	Days postfertilization
EGFR	Epidermal growth factor receptor
PDL1	Programmed death ligand 1
APS	Ammonium persulphate
DEPC	Diethyl pyrocarbonate
Mo-AS	Monovalent-AS1411
RIPA	Radio immunoprecipitation assay
BCA	Bicinchoninic acid assay
TBST	Tris-borate-sodium Tween-20
ECL	Electrochemiluminescence
HRP	Horseradish peroxidase
DAB	Diaminobenzidine
DiO	3,3′-dioctadecyloxacarbocyanine perchlorate
FAS	Factor-related Apoptosis
