# Supplementary material for: DNA Tweezers with Replaceable Clamps for the Targeted Degradation of Cell Membrane Proteins
Source: Pharmaceutics. 2025 Jun 17;17(6):785. doi: 10.3390/pharmaceutics17060785 (PMC12197088; doi:10.3390/pharmaceutics17060785)
Supplement: Supplementary file 1 [file pharmaceutics-17-00785-s001.zip › pharmaceutics-3607652-supplementary.pdf]

Supplementary Materials

Table S1. DNA Sequences used in this work

Name	Sequence (5'→3')
IGFIIR aptamer	GGGCGCGTAGATGACGAGCAGTCCTAACATCGTTTAGGAC
NCL aptamer (AS1411)	GGTGGTGGTGGTTGTGGTGGTGGTGG
EGFR aptamer	GGACGGATTTAATCGCCGTAGAAAAGCATGTCAAAGCCGGAACCGTCCCGACGACGACGACGACGACGA
PDL1 aptamer	TAAAGGGCGGGGGGTGGGGTGGTTGGTAGTTGTTTTTTCTGTTTC
Nucleic acid module 1	TTTTTTTTTTTTTTT/Symmetric/C12/GGCGCGTAGATGACGAGCAGTCCTAACATCGTTTAGGAC
polyT-IGFIIR	TTTTTTTTTTTTTTTGGGCGCGTAGATGACGAGCAGTCCTAACATCGTTTAGGAC
FAM-polyT-IGFIIR	TTTTTTTTTTTTTTTGGGCGCGTAGATGACGAGCAGTCCTAACATCGTTTAGGAC-FAM
Nucleic acid module 2(target NCL)	AAAAAAAAAAAAAAAGGTGGTGGTGGTTGTGGTGGTGGTGG-FAM
Nucleic acid module2(target EGFR)	AAAAAAAAAAAAAAAGGACGGATTTAATCGCCGTAGAAAAGCATGTCAAAGCCGGAACCGTCCCGACGACGACGACGACGACGA
Nucleic acid module 2(target PDL1)	AAAAAAAAAAAAAAATAAAGGGCGGGGGGTGGGGTGGTTGGTAGTTGTTTTTTCTGTTTC
FAM-Nucleic acid module 1	TTTTTTTTTTTTTTT/Symmetric//C12/GGGCGCGTAGATGACGAGCAGTCCTAACATCGTTTAGGAC-FAM
Cy5-Nucleic acid module 1	TTTTTTTTTTTTTTT/Symmetric//C12/GGGCGCGTAGATGACGAGCAGTCCTAACATCGTTTAGGAC-Cy5
Cy5-Nucleic acid module 2(target NCL)	AAAAAAAAAAAAAAAGGTGGTGGTGGTTGTGGTGGTGGTGG-Cy5
Cy5-Nucleic acid module 2(target EGFR)	AAAAAAAAAAAAAAAGGACGGATTTAATCGCCGTAGAAAAGCATGTCAAAGCCGGAACCGTCCCGACGACGACGACGACGACGA-Cy5

Cy5-Nucleic acid module 2(target PDL1)	AAAAAAAAAAAAAAATAAAGGGCGGGGGGTGGGGTGGTTGGTAGTTGTTTTTTCTGTTTC-Cy5
Cy5-Library	NNNNNNNNNNNNNNNNNNNNNNNNNNNNNNNNNNNNNNNNNNNNNNNNNNNNN-Cy5

C12 represent a linear alkyl chain configuration comprising twelve carbon atoms; Symmetric means the base before here is double sequence (15 poly T is double sequence); FAM is a fluorescein which can be excitation at 494 nm; Cy5 is a dye with excitation designed for use with the 633 nm or 647 nm laser lines.

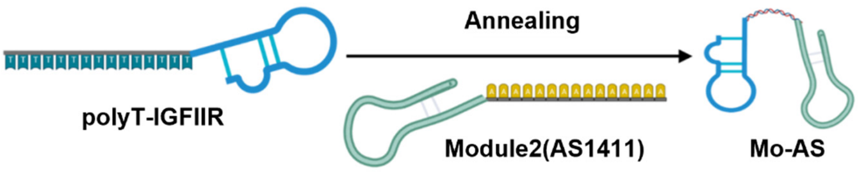

Figure S1. Synthetic strategy of Mo-AS.

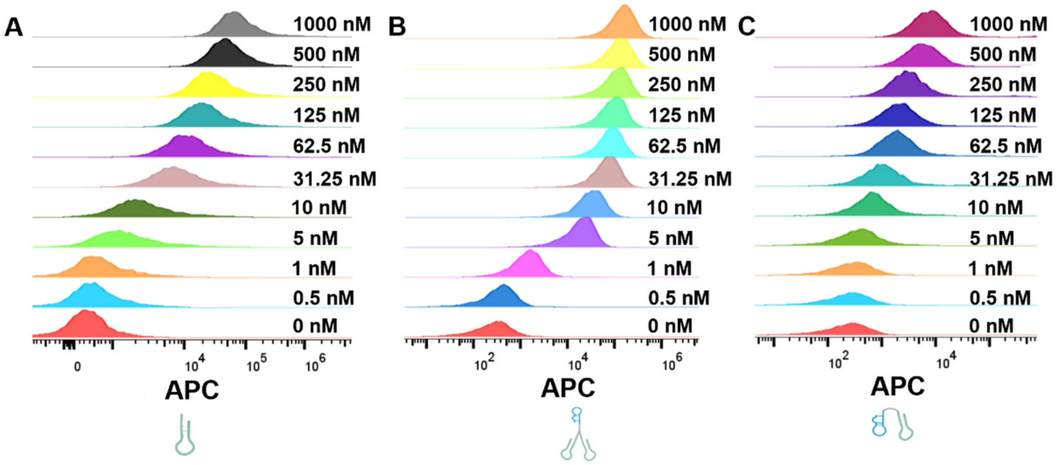

Figure S2. Binding affinity of AS1411 (A), DNA tweezer (B), and Mo-AS (C) to MCF-7 cells were studied with Flow cytometric.

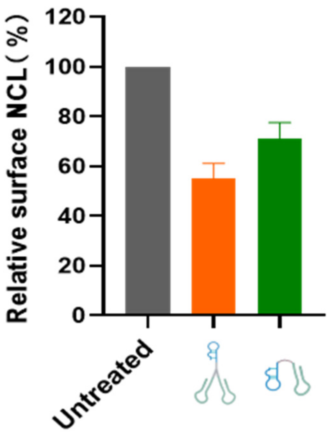

Figure S3. MCF-7 cells were treated with DNA tweezer or Mo-AS for 3h, protein level of NCL was studied with flow cytometry, and the fluorescence intensity change was concert into protein concentration change. The data are shown as means ± SDs (n = 2).

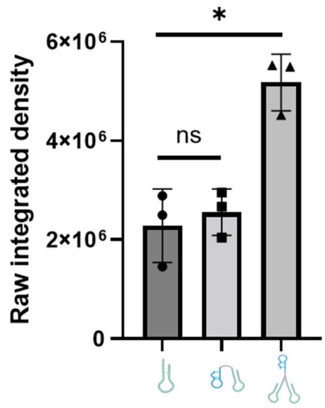

Figure S4. MCF-7 tumor-bearing mice were intravenously injected with Cy5-labeled AS1411, Mo-AS or DNA tweezer, tumor tissues were collected and tissue sections were prepared for confocal imaging. The intensity of residual Cy5 fluorescence on tumor tissue is shown for each group. The data are shown as means ± SDs (n = 3). *p < 0.05; ns, not significant.

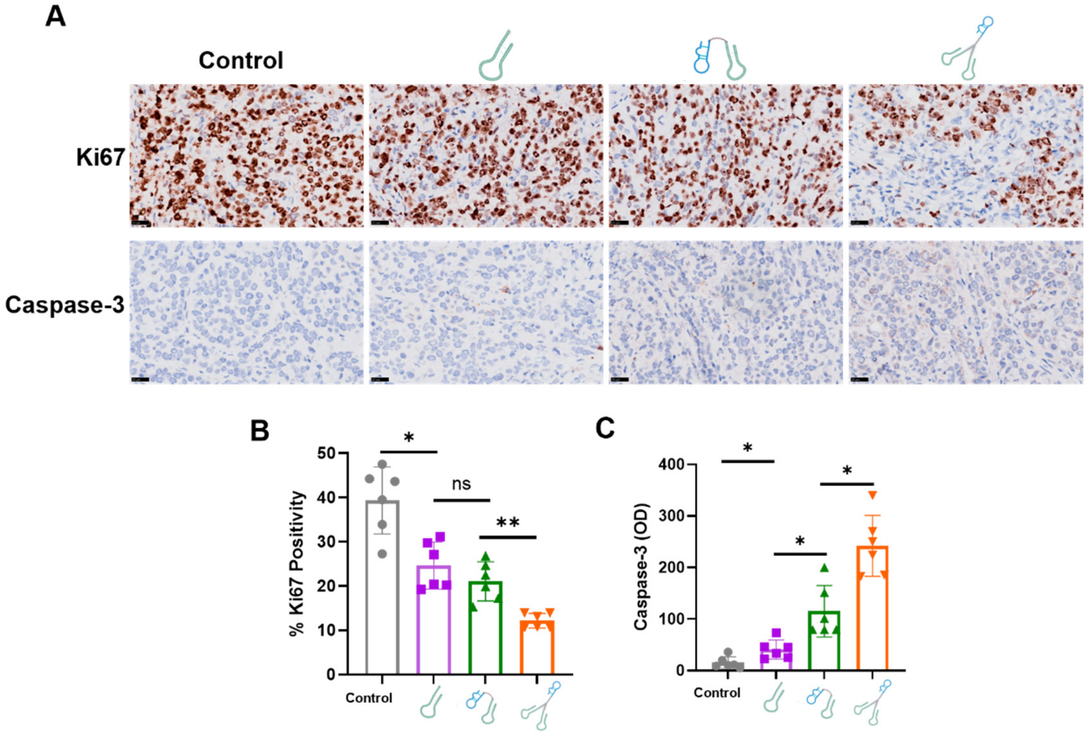

Figure S5. (A) IHC stain of Ki67 and Caspase-3 in dissected tumor tissues. Scale bar: 25 μm (B) Quantification of Ki67-positive cells in tumors (3 tumors per group). For quantification, analysis of a total of 2 fields per tumor was performed. *p < 0.05, **p < 0.01; ns, not significant. (C) Quantification of the optical density values of Caspase-3 in tumors (3 tumors per group). For quantification, analysis of a total of 2 fields per tumor was performed. *p < 0.05.

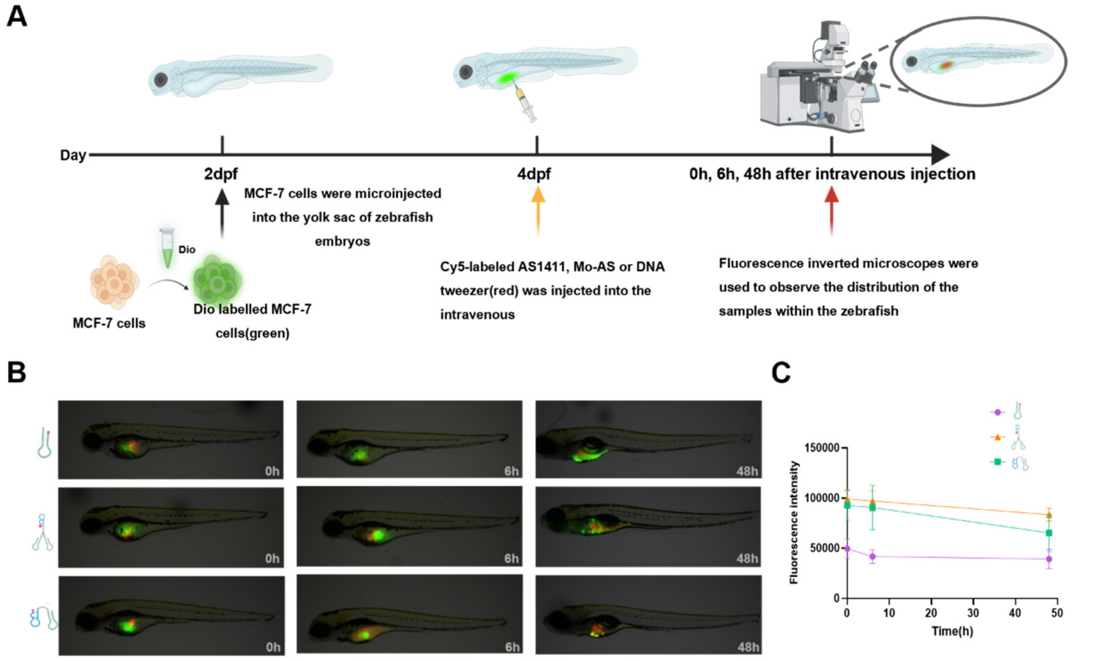

Figure S6. (A) Schematic illustration for exploring the distribution of AS1411, DNA tweezer and Mo-AS in zebrafish over time. (B) MCF-7 tumor xenograft zebrafish model was constructed and Cy5-labeled AS1411, Mo-AS, and DNA tweezer (red) were injected via intravenous respectively, then imaging was performed with fluorescence microscopy. Tumor cells were labeled with green fluorescence. (C) Fluorescence values for each group on tumor sections. The data are shown as means ± SDs (n = 10).

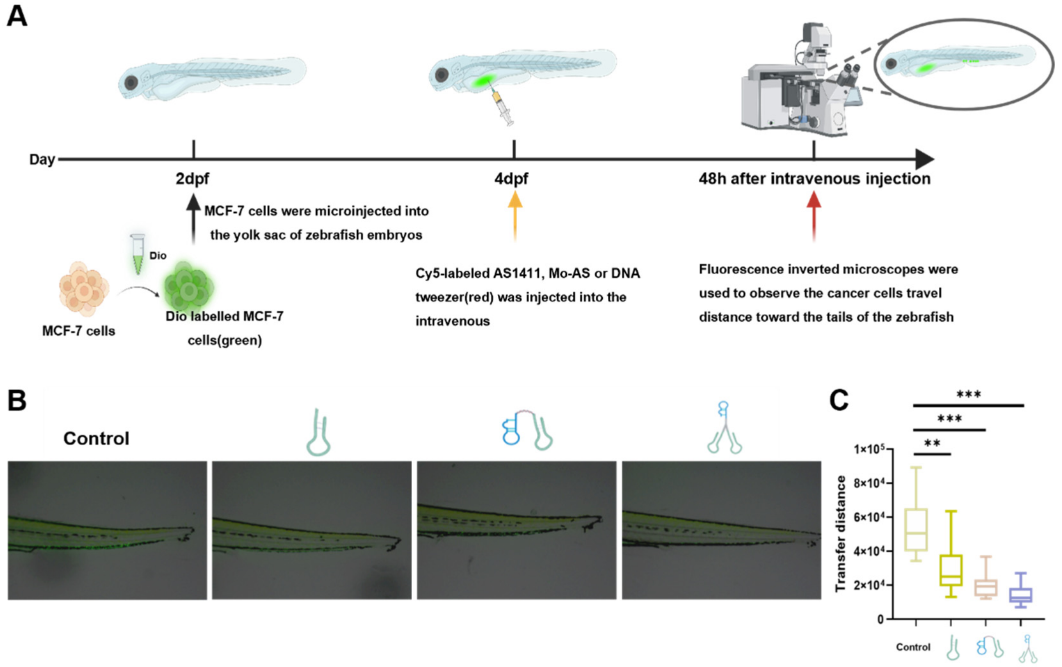

Figure S7. (A) Schematic illustration for exploring the inhibition of tumor(green) metastasis in zebrafish by AS1411, Mo-AS or DNA tweezer. (B) Representative image of the metastatic distance of tumor cells

to the tail at 48 h post-injection. (C) Transfer distance of MCF-7 cells in the tail of zebrafish embryos 48 h after injection. The data are shown as means ± SDs (n = 10). **p < 0.01, ***p < 0.001.

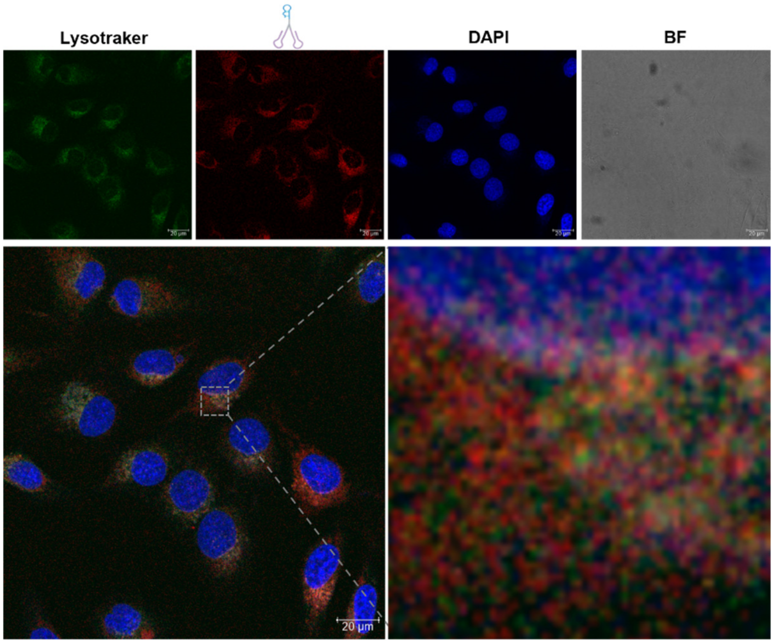

Figure S8. Confocal images showing cellular uptake and lysosomal localization of EGFR-DNA tweezer in Hela cells. Scale bar: 20 μm

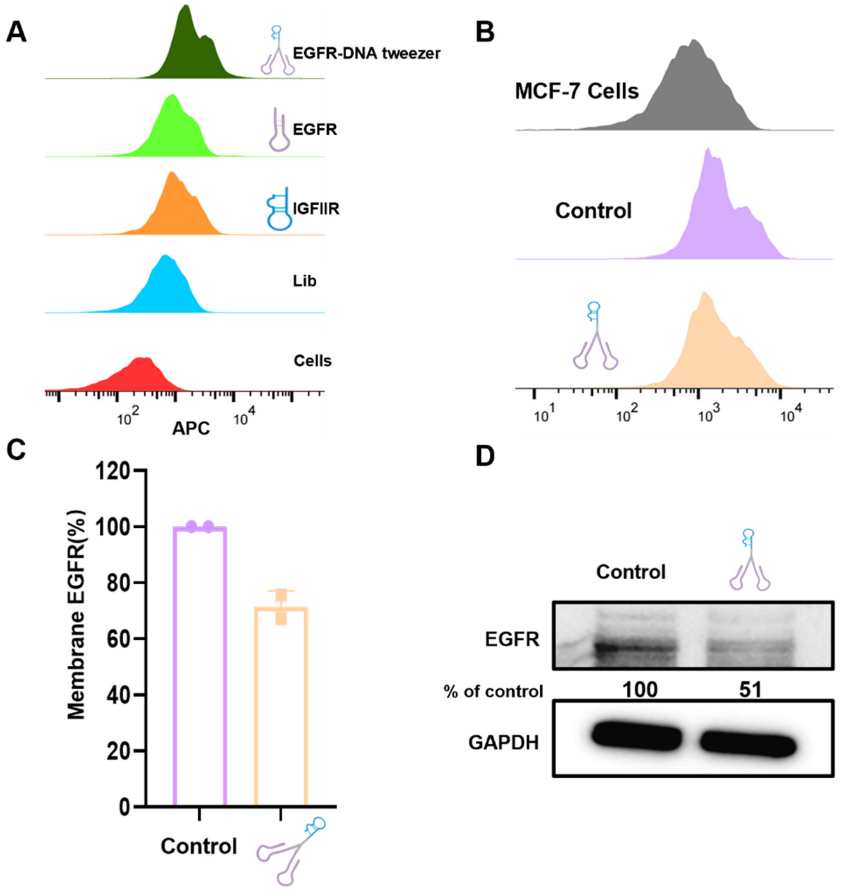

Figure S9. (A) Binding ability of EGFR-DNA tweezer to MCF-7 cells was tested with flow cytometry. Library, IGFIIR-aptamer, EGFR-aptamer were used as control. (B) Cellular surface EGFR was studied with anti-EGFR antibody by flow cytometry after treatment with EGFR-DNA tweezer. (C) The fluorescence intensity of EFGR antibody was quantified. The data are shown as means ± SDs (n = 2). (D) EGFR protein level in MCF-7 cells after treatment with EGFR-DNA tweezer was verified by Western Blotting.

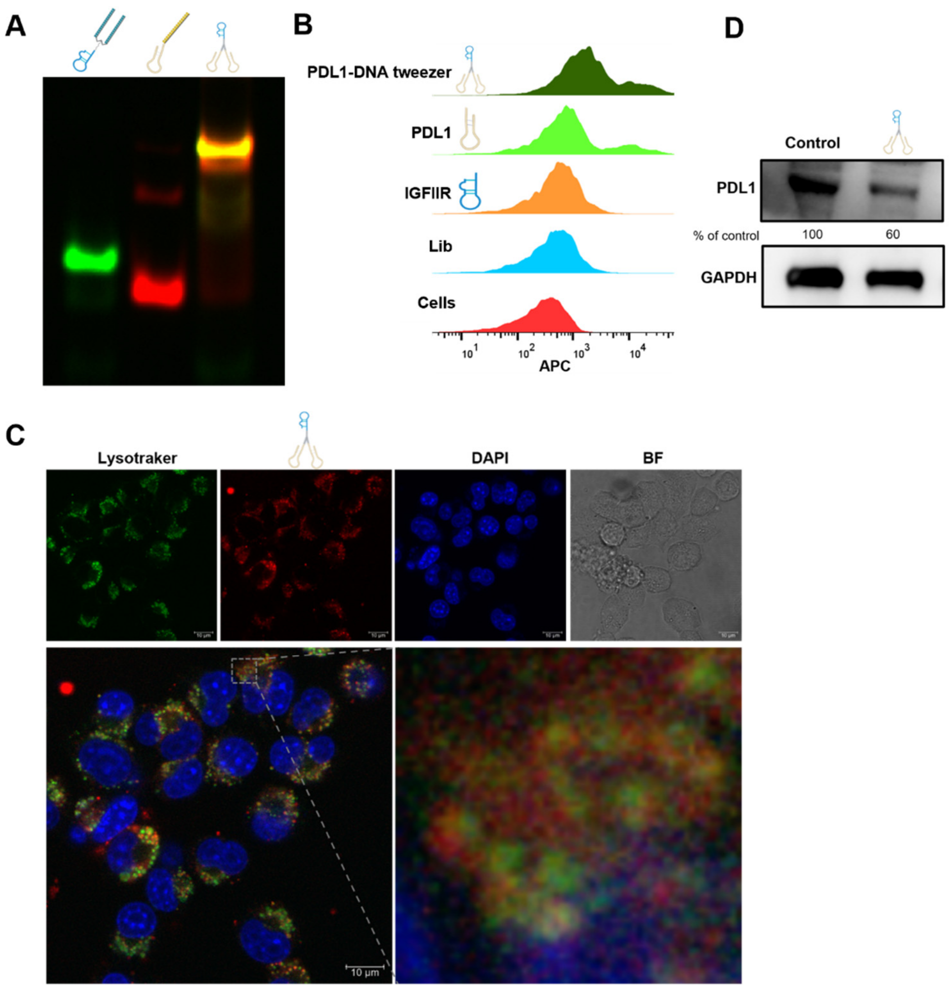

Figure S10. (A) The assemble of PDL1 targeted DNA tweezer was verified with native polyacrylamide gel. Lane1: FAM labeled-module1; Lane2: Cy5 labeled-module2 (target PDL1); Lane3: Cy5, FAM labeled-DNA tweezer. (B) Binding ability of PDL1-DNA tweezer to 4T1 cells was tested with flow cytometry. Library, IGFIIR-aptamer, PDL1-aptamer were used as control. (C) Confocal images showing cellular uptake and lysosomal localization of PDL1-DNA tweezer in 4T1 cells. Scale bar: 10 μm. (D) PDL1 protein level of 4T1 cells after treatment with PDL1-DNA tweezer was verified by Western Blotting.

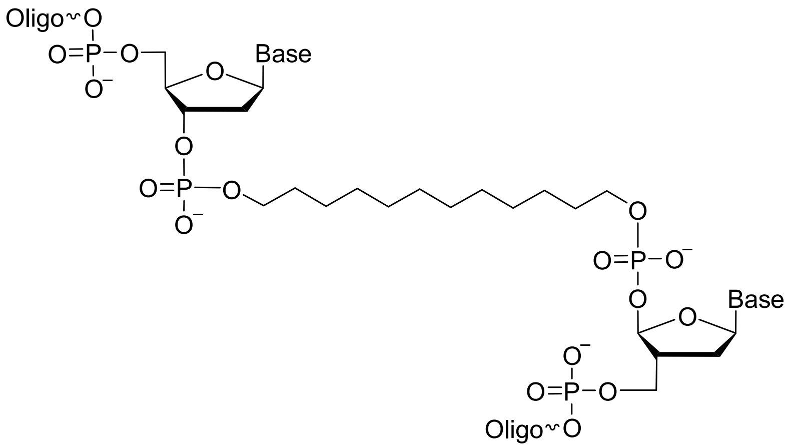

Figure S11. The structure of C12.
